# Intrafusal cross‐bridge dynamics shape history‐dependent muscle spindle responses to stretch

**DOI:** 10.1113/EP090767

**Published:** 2023-07-10

**Authors:** Surabhi N. Simha, Lena H. Ting

**Affiliations:** ^1^ Wallace H. Coulter Department of Biomedical Engineering Emory University and The Georgia Institute of Technology Atlanta Georgia USA; ^2^ Department of Rehabilitation Medicine, Division of Physical Therapy Emory University Atlanta Georgia USA

**Keywords:** muscle spindle firing, sensory encoding, short‐range stiffness

## Abstract

Computational models can be critical to linking complex properties of muscle spindle organs to the sensory information that they encode during behaviours such as postural sway and locomotion where few muscle spindle recordings exist. Here, we augment a biophysical muscle spindle model to predict the muscle spindle sensory signal. Muscle spindles comprise several intrafusal muscle fibres with varied myosin expression and are innervated by sensory neurons that fire during muscle stretch. We demonstrate how cross‐bridge dynamics from thick and thin filament interactions affect the sensory receptor potential at the spike initiating region. Equivalent to the Ia afferent's instantaneous firing rate, the receptor potential is modelled as a linear sum of the force and rate change of force (yank) of a dynamic bag1 fibre and the force of a static bag2/chain fibre. We show the importance of inter‐filament interactions in (i) generating large changes in force at stretch onset that drive initial bursts and (ii) faster recovery of bag fibre force and receptor potential following a shortening. We show how myosin attachment and detachment rates qualitatively alter the receptor potential. Finally, we show the effect of faster recovery of receptor potential on cyclic stretch–shorten cycles. Specifically, the model predicts history‐dependence in muscle spindle receptor potentials as a function of inter‐stretch interval (ISI), pre‐stretch amplitude and the amplitude of sinusoidal stretches. This model provides a computational platform for predicting muscle spindle response in behaviourally relevant stretches and can link myosin expression seen in healthy and diseased intrafusal muscle fibres to muscle spindle function.

## Abstract (highlights)

- **What is the central question of the study?**
  A computational biophysical muscle model was used to ask how muscle cross‐bridge dynamics shape the information that can be encoded by intrafusal muscle fibres within the muscle spindle.
- **What is the main finding and its importance?**
  Both actin and myosin dynamics and their interactions can shape muscle spindle sensory signals and are necessary to simulate history‐dependent muscle spindle firing properties to be more in line with experimental observations. The tuned muscle spindle model shows that non‐linear and history‐dependent muscle spindle firing properties to sinusoids reported previously emerge from intrafusal cross‐bridge dynamics.

## 1 INTRODUCTION

Computational biophysical models can provide critical insight on the role of the sensory function of muscle spindles in behaviour. Present in most mammalian skeletal muscles, muscle spindles consist of specialized intrafusal muscle fibres, whose properties and activation shape the temporal pattern and amplitude of muscle spindle firing when the muscle is stretched. Mechanotransduction in muscle spindle group Ia afferents occurs in the sensory endings wrapped around the central encoding region of the intrafusal fibres, generating receptor potentials driving muscle spindle firing, typically measured in acute preparations during imposed muscle stretch (Boyd, 1962; Hulliger, 1984; Prochazka, 2021). Muscles are continually stretched and shortened during behaviours that involve cyclic movements such as postural sway or locomotion. However, measuring muscle spindle response during behaviour in vivo or even in vitro is extremely challenging. Therefore, computational models can be key to understanding how muscle spindles function in behaviours. Prior muscle spindle models represent the intrafusal muscle fibre using some variation of phenomenological Hill‐type muscle models (Hasan, 1983; C.‐C. K. Lin & Crago, 2002; Schaafsma et al., 1991) that generalize poorly to unsteady behaviours. Further, intrafusal muscle fibres have varied contractile properties that play a key role in shaping muscle spindle firing characteristics (Boyd, 1976; Poppele & Quick, 1985, 1981). Biophysical muscle spindle models simulating cross‐bridge dynamics of the intrafusal fibres can simulate intrafusal muscle fibre stretch leading to Ia afferent firing during unsteady and cyclic behaviours (Campbell & Moss, 2002).

Although our recent muscle spindle model demonstrated that multiple properties of muscle spindle firing emerged from muscle cross‐bridge dynamics of two intrafusal fibres, its history‐dependent force recovery was too slow to predict cyclic movements at frequencies relevant to postural and locomotor behaviours. Blum et al. found that the receptor potential of the Ia afferent could be modelled as a weighted sum of the muscle force and yank in passive muscle stretch where intrafusal and extrafusal muscle force are assumed to have similar but scaled time histories (Blum et al., 2017). Based on that finding, Blum et al. then developed a muscle spindle model consisting of two intrafusal fibres, broadly representing those generating more rapid, dynamic responses to stretch velocity and accelerations such as the bag1 fibres, and those generating more static responses to muscle length changes, such as the bag2 and chain fibres based on different muscle cross‐bridge properties (Blum et al., 2020; Huxley, 1957). Receptor potentials were modelled as a scaled and weighted sum of the force from the chain, and the force and time‐rate of change of force, termed yank, from the bag. This model predicted many properties of muscle spindle firing, such as the initial burst and its magnitude varying with ramp velocity due to the short‐range stiffness of the bag fibre, and history‐dependent properties of the muscle spindle response – the initial burst disappeared on the second of two consecutive triangular stretch–shorten cycles but returned as the inter‐stretch‐interval was increased. However, the recovery time of the initial burst of ∼3 s was much slower than the experimentally observed recovery time of 0.5 s (Proske & Gregory, 1977). Thus, the model was unable to predict realistic cyclic behaviours such as postural sway and movement as they involve repeated stretch–shorten cycles.

However, Blum et al. (2020) only modelled myosin dynamics of the thick filament but did not include thin filament actin dynamics, which may explain why their predictions of muscle spindle responses were not within physiological time scales for cyclic behaviours. They used a myosin attachment–detachment model where fixed rates of attachment and detachment were selected for the bag and chain fibres. The number of active cross‐bridges, which determines the force that the intrafusal fibres develop on the central encoding region, was determined based on muscle fibre activation level, bypassing actin dynamics of the thin filament. However, actin–myosin interactions between the thick and thin filaments also affect cross‐bridge dynamics and play an important role in the rate of force development through a property known as interfilament cooperativity (K. Campbell, 1997). Such actin–myosin interactions have been modelled and validated on an open‐source platform, MATMyoSim, that hosts a number of biophysical muscle models. We used the latest ‘two‐state cross‐bridge muscle model’ from MATMyoSim and combined it with the receptor potential model from Blum et al., to characterize the role of the thin filament interaction with the thick filament in the bag and chain fibres, and to enable more physiological muscle spindle responses in cyclic behaviours.

Here our goal was to include actin dynamics in the biophysical muscle spindle model (Blum et al., 2020) to improve predictions of history‐dependent muscle spindle firing in cyclic behaviours. Importantly, the inclusion of thin filament, allows us to more closely model experiments by using the concentration of calcium in the intracellular matrix to simulate the level of activation of the intrafusal fibres, rather than explicitly controlling the fraction of cross‐bridges that are active. Using MATMyoSim, we characterized how adding components of the thin filament alter bag and chain responses to a ramp‐and‐hold stretch, altering their contributions to the receptor potential. We then varied the myosin attachment and detachment rate functions of the intrafusal fibres to show how these can shape the receptor potential dynamic response. Then we selected an intrafusal fibre model that matches a muscle spindle firing rate whose qualitative features are comparable to that observed empirically from a rat soleus muscle from Housley et al. (2023). We then used that model to predict the non‐linear and history‐dependent receptor potential in response to three behaviourally relevant stretch–shorten protocols.

## 2 METHODS

### 2.1 Structure of the muscle spindle model

**FIGURE 1:**
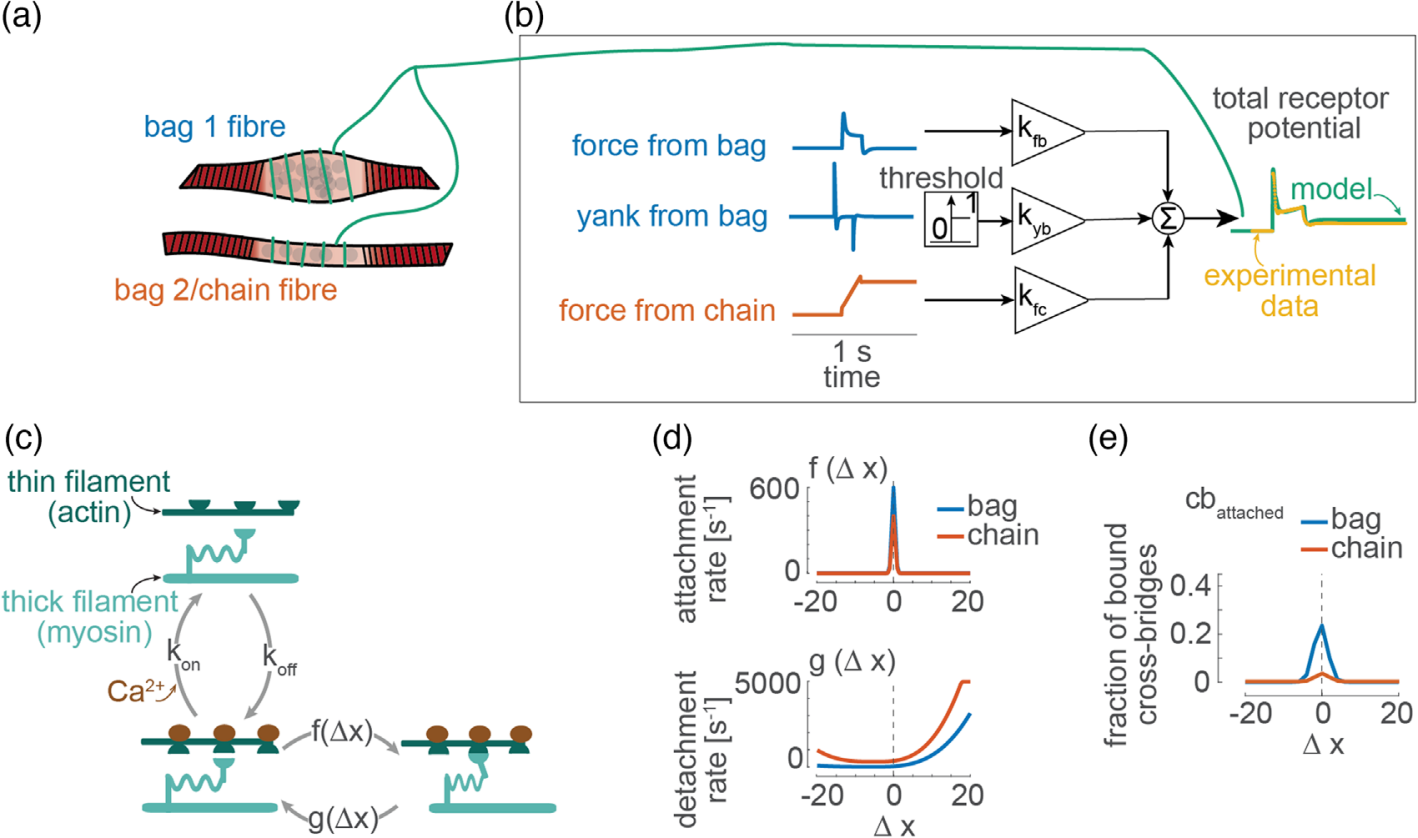
Muscle spindle model. (a) The muscle spindle is modelled as consisting of a bag1 and a bag 2/chain fibre. (b) The receptor potential at the spike initiating region of the Ia afferent is modelled using a phenomenological receptor potential model based on Blum et al. (2020). (c) The *bag* and *chain* intrafusal fibres are modelled using a cross‐bridge muscle model with two myosin states of the thick filament (bottom left and right) and two actin states of the thin filament (left top and bottom). *k*_on_ and *k*_off_ represent the activation and deactivation rates of the actin sites respectively. (d) Cross‐bridges are formed or lost when myosin attaches and detaches from actin according to the attachment rate f(Δx) and detachment rate g(Δx). These rate functions primarily drive the functional difference between the bag and chain fibres. (e) Since a cross‐bridge is modelled as a spring, the magnitude of force generated by the intrafusal fibres at any given time is determined by the fraction of bound cross‐bridges and their length.

Following the structure of Blum et al. (2020), we simulated two intrafusal muscle fibres acting in parallel, a fast chain fibre and a slower bag fibre, with a receptor potential model (Figure 1). We assumed that the fibre contractile properties are uniform throughout their length. Together, they generate the receptor potential in one muscle spindle Ia afferent. The model does not explicitly represent the mechanotransduction process between the intrafusal muscle fibre and the sensory afferent. Rather, we used a phenomenological model of the process, whereby the resulting receptor potential is computed as a weighted sum of the force from the intrafusal fibres and time rate of change of the force from the bag fibre (Blum et al., 2020). To study the effect of the intrafusal fibres on the Ia afferent output, we kept all the parameters of the receptor potential model fixed for all simulations. We did not model the spiking behaviour of the Ia afferent here, but we expect it to qualitatively match the receptor potential (Hunt & Ottoson, 1975, 1976).

### 2.2 Phenomenological receptor potential model

We used the receptor potential model from Blum et al. to convert the forces from the bag and chain fibres directly to the receptor potential of the Ia afferent. While intrafusal fibres can morphologically be classified into many groups, they may still be structurally and functionally classified as either a slow‐twitch, dynamic bag1 fibre or a fast‐twitch, static bag2 and chain fibre (Banks et al., 1977; Thornell et al., 2015). For simplicity, we modelled a *bag* fibre to represent the dynamic bag1 fibres and the *chain* fibre to represent both the static bag2 and static chain fibres together, similar to prior models (Hasan, 1983; Schaafsma et al., 1991). The bag component of the receptor potential is computed as the weighted sum of the force and half‐wave‐rectified time derivative of force, termed yank, from the bag fibre. The yank is half‐wave rectified to model the dynamic part of the receptor current as encoding only the positive rate of change of force. The chain component of the receptor potential is computed as a weighted force from the chain fibre. The total receptor potential of the Ia afferent is the sum of these two components:

(1) $$\begin{array}{c} r_{\mathrm{total}}\left(t\right)=\left(k_{f_{c}}\times F_{c}\left(t\right)\right)+\left(k_{f_{b}}\times F_{b}\left(t\right)\right)+\left(k_{y_{b}}\times \frac{dF_{b}\left(t\right)}{dt}\right) \end{array}$$

This is scaled by a factor of 2 × 10^5^ to account for the transformation from force signals to receptor potential signals. For all results presented here the weighting on the force from the bag ($k_{f_{b}}$) is 0.4, and yank from the bag ($k_{y_{b}}$) is 0.005 and from the force from the chain ($k_{f_{c}}$) is 0.5, also given in Table 1. For the scaling factors we assume arbitrary units that match a conversion from the units of force to units of current.

**TABLE 1:** List of model parameters divided by receptor potential model and intrafusal fibre model.

Description	Symbol	Value	Units
Receptor potential model properties			
Weighting on force from bag	$k_{f_{b}}$	0.4	a.u.
Weighting on force from chain	$k_{f_{c}}$	0.5	a.u.
Weighting on yank from bag	$k_{y_{b}}$	0.005	a.u.
Intrafusal fibre model: common properties			
Concentration of calcium ions; representing activation	Ca^2 +^	10^−6.4^	M
Activation rate constant	*k*_on_	8 × 10^7^	M^−1^ s^−1^
Deactivation rate constant	*k*_off_	200	s^−1^
Length of thin filament	length_thin filament_	1120	nm
Resting length of half‐sarcomere	*L*_0_	1300	nm
Length of thin filament	length_thick filament_	815	nm
Length of bare zone	length_bare zone_	80	nm
Number of cross‐bridges per unit area	cb_density_	6.9 x 10^16^	m^−2^
Stiffness of a cross‐bridge	cb_stiffness_	0.001	pN nm^−1^
Thermal energy constant of myosin heads	const_f_	7.2 x 10^−2^	pN nm
Intrafusal bag fibre model properties			
Offset in detachment rate function for bag fibre	offset_bag_	0.5	s^−1^
Passive tissue stiffness in bag fibre	passive_stiffness, bag_	90	N m^−2^ nm^−1^
Slack length of bag fibre	length_slack, bag_	1050	nm
Intrafusal chain fibre model properties			
Offset in detachment rate function for chain fibre	offset_chain_	10	s^−1^
Passive tissue stiffness in chain fibre	passive_stiffness, chain_	250	N m^−2^ nm^−1^
Slack length of chain fibre	length_slack, chain_	1200	nm

All values listed here were held constant through all simulations.

### 2.3 Biophysical model of intrafusal muscle fibres

We modelled an intrafusal fibre as a single half sarcomere whose force output is governed by cross‐bridge dynamics (Figure 1). For reproducibility and open‐access, we implemented our model with MATMyoSim (https://campbell‐muscle‐lab.github.io/MATMyoSim/). Derived from the original Huxley muscle model equations (Huxley, 1957), it models a half‐sarcomere as a thin and thick filament consisting of a fixed number of actin sites and myosin heads, respectively.

Actin sites can be activated or deactivated, and the number of activated actin at any time (Actin_activated_(*t*)) is governed by calcium concentration ($Ca^{2+}\left(t\right)$), overlap between the thin and thick filaments ($n_{\mathrm{overlap}}\left(t\right)$, Equation 3), activation rate (*k*_on_), deactivation rate (*k*_off_) and cooperativity (*k*_coop_) (Equation 2), where cooperativity determines the myosin‐dependent activation of actin sites (Campbell, 2014). All calculations are in fractions and scaled by number of cross‐bridges per unit area (cb_density_) at the end, assuming uniform distribution.

(2) $$\begin{array}{ccc} & & \frac{\mathrm{dActi}n_{\mathrm{activated}}\left(t\right)}{dt}=k_{\mathrm{on}}\cdot {\mathrm{Ca}}^{2+}\left(t\right)\cdot \left(n_{\mathrm{overlap}}\left(t\right)-\mathrm{Acti}n_{\mathrm{activated}}\left(t\right)\right)\\ & & \cdot \;\left(1+k_{\mathrm{coop}}\cdot \frac{\mathrm{Acti}n_{\mathrm{activated}}\left(t\right)}{n_{\mathrm{overlap}}\left(t\right)}\right)-k_{\mathrm{off}}\cdot \left(\mathrm{Acti}n_{\mathrm{activated}}\left(t\right)-{\mathrm{cb}}_{\mathrm{attached}}\left(t\right)\right)\\ & & \cdot \;\left(1+k_{\mathrm{coop}}\cdot \left(\frac{n_{\mathrm{overlap}}\left(t\right)-\mathrm{Acti}n_{\mathrm{activated}}\left(t\right)}{n_{\mathrm{overlap}}\left(t\right)}\right)\right) \end{array}$$

where

(3) $$n_{\mathrm{overlap}}\left(t\right)=\frac{\mathrm{lengt}h_{\mathrm{thin}\;\mathrm{filament}}-\left(\mathrm{lengt}h_{\mathrm{half}\;\mathrm{sarcomere}}\left(t\right)-\mathrm{lengt}h_{\mathrm{thick}\;\mathrm{filament}}\right)}{\mathrm{lengt}h_{\mathrm{thick}\;\mathrm{filament}}-\;\mathrm{lengt}h_{\mathrm{bare}\;\mathrm{zone}}}$$

where length_half sarcomere_ is the end‐to‐end length of the half sarcomere at *t* and the remaining lengths are constants given in Table 1. For our intrafusal fibre model the activation represents the effects of intrafusal gamma motor neuron drive to intrafusal fibres.

Myosin heads can exist in one of two states, attached or detached, where attached heads form cross‐bridges. The transition between these states is governed by the number of activated actin sites Actin_activated_(*t*), strain‐dependent attachment rate function (f(Δx)) and strain‐dependent detachment rate function (g(Δx)):

(4) $$\begin{array}{ccc} \frac{\delta \mathrm{Myosi}n_{\mathrm{attached}}\left(\Delta x,t\right)}{\delta t} & = & f\left(\Delta x\right)\cdot \mathrm{Myosi}n_{\mathrm{detached}}\left(t\right)\\ & & \cdot \;\left(\mathrm{Acti}n_{\mathrm{activated}}\left(t\right)-cb_{\mathrm{attached}}\left(t\right)\right)-g\left(\Delta x\right)\\ & & \cdot \;\mathrm{Myosi}n_{\mathrm{attached}}\left(\Delta x,t\right) \end{array}$$

(5) $$\begin{array}{c} \frac{\delta \mathrm{Myosi}n_{\mathrm{detached}}\left(t\right)}{\delta t}=\int_{-\infty }^{\infty }\begin{array}{c} g\left(\Delta x\right)\cdot \mathrm{Myosi}n_{\mathrm{attached}}\left(\Delta x,t\right)dx\\ -\int_{-\infty }^{\infty }f\left(\Delta x\right)\cdot \mathrm{Myosi}n_{\mathrm{detached}}\left(t\right)dx \end{array}\; \end{array}$$

where $cb_{\mathrm{attached}}\left(t\right)\;=\;\sum_{i}\mathrm{Myosi}n_{\mathrm{attached},i}$ is the total number of bound cross‐bridges at time *t*. We chose the shape of the rate functions (Figure 1d) to match literature (Blum et al., 2020; Campbell, 2014) and only tuned one parameter for each – slope of the attachment curve (*b*_f_ for bag and *c*_f_ for chain) and vertical offset of the detachment curve (*b*_g_ and *c*_g_). The following equations are for the bag fibre but the chain fibre follows the same shape:

(6) $$\frac{df_{\mathrm{bag}}\left(\Delta x\right)}{dx}=b_{f}\;e^{\frac{-cb_{\mathrm{stiffness}}\cdot \Delta x^{2}}{\mathrm{cons}t_{f}}}$$

(7) $$\begin{array}{c} \;\frac{dg_{\mathrm{bag}}\left(\Delta x\right)}{dx}=\left\{\begin{array}{c} b_{g}+\left|0.2\cdot {\left(\Delta x+5\right)}^{3}\right|\;\mathrm{for}\;\Delta x<-5\\ b_{g}+\left|0.3\cdot {\left(\Delta x+5\right)}^{3}\right|\;\mathrm{for}\;\Delta x\geq -5 \end{array}+\mathrm{offse}t_{\mathrm{bag}}\right. \end{array}$$

While structural parameters such as sarcomere length have been measured from intrafusal fibres (Banks, 2005; Poppele & Quick, 1981), much of the literature on functional parameters such as cross‐bridge stiffness or attachment rates is from myosin types more prevalent in extrafusal fibres (Veigel et al., 2003). Thus, for all parameters, we used constant default values from either MATMyoSim or from Blum 2020 (Table 1). Only *k*_coop_, *b*_f_, *c*_f_, *b*_g_ and *c*_g_ were tuned to study their effects on the receptor potential, as described in the following sections.

The total force from each half sarcomere is the sum of forces from all cross‐bridges and non‐contractile parallel passive tissue modelled as a parallel elastic element. We did not model any extracellular matrix here. The cross‐bridge was modelled as a spring such that the force from each one depends on its length Δx and a constant cross‐bridge stiffness (cb_stiffness_, Equation 8). We estimate and report stress – force per unit area of cross‐section – as the output from the intrafusal fibre model. Equation (8) can be applied to the chain fibre by replacing the fibre‐specific values with those of the chain fibre.

(8) $$\begin{array}{ccc} \mathrm{sarcomere}\;\mathrm{stress}\left(t\right)\; & = & \;1\times 10^{-9}\cdot {\mathrm{cb}}_{\mathrm{density}}\cdot \sum_{i}{\mathrm{cb}}_{\mathrm{stiffness}}\cdot \Delta x_{i}\\ & & \cdot \;{\mathrm{cb}}_{\mathrm{attached},i}\left(t\right)\;+\;\mathrm{passiv}e_{\mathrm{stiffness},\;\mathrm{bag}}\\ & & \cdot \;\left(\mathrm{lengt}h_{\mathrm{half}\;\mathrm{sarcomere}}\left(t\right)-\mathrm{lengt}h_{\mathrm{slack},\;\mathrm{bag}}\right)\;\; \end{array}$$

Assuming a uniform cross‐sectional area, this stress is converted to force by the scaling factors with arbitrary units ($k_{f_{b}}$, $k_{f_{c}}$) when it is input to the receptor potential model. A simulation begins with the intrafusal muscle model receiving, as input, a time series of length changes and a time series of calcium concentration. Calcium concentration is reported in units of pCa – an activation of 0–100% maps onto a sigmoidal curve from pCa 9 to pCa 4.5 (Campbell, 2014). Once the forces from the intrafusal fibres have been estimated for the entire protocol, they are passed onto the receptor potential model.

To understand the effect of cross‐bridge dynamics on receptor potential, which is directly driven by cross‐bridge force, we divided cross‐bridge force generation into two broad mechanisms: (i) thin filament effects – actin activation, deactivation and cooperativity, and (ii) thick filament effects – myosin attachment and detachment rates. While only the thin filament effects are an addition relative to the Blum 2020 model, we describe the effects of both here since they affect each other.

### 2.4 Effect of thin filament dynamics on force response to stretch

We broke down the effect of thin filament dynamics as arising from three mechanisms.

#### 2.4.1 Calcium‐based activation of actin sites

First, the calcium concentration and the activation rate constant (*k*_on_, Equation 2) determine the number of active actin sites available for binding and the rate at which they become available. We expect this to slow down the rate of force development during activation but since all our simulations here were performed after activation has reached steady‐state, we did not observe this effect in the figures. We still chose to use a calcium concentration to specify activation so that the model is generalizable to conditions of changing activation. A pCa concentration is a measure of the number of moles of calcium ions in a solution and therefore serves as a means to specify the number of calcium ions available in the intracellular matrix with which the actin molecules can react. We used a low calcium concentration of pCa 6.4 to simulate near passive stretches since the experimental data we compared our model to were from an anaesthetized animal (refer section: ‘Tuning model parameters to match experimental data’). Note that the relationship between pCa and activation depends on the filament properties and therefore differs as we introduce new properties of the thin filament here. For simplicity, we maintained a constant pCa of 6.4 across all conditions, corresponding to ∼10% activation of the chain fibre in the final model used to predict history‐dependence.

#### 2.4.2 Deactivation of actin sites

Second, we included a rate of deactivation of actin sites (*k*_off_) to represent the cycling of calcium ions after power stroke. We expect a non‐linear effect of this on the force since it places a time‐dependent limit on the rate at which myosin heads can bind.

#### 2.4.3 Inter‐filament cooperativity

Third, we included a phenomenon of cooperativity observed between the thin and thick filament where the presence of attached myosin heads can increase the number of actin sites available for binding (Campbell, 1997) using *k*_coop_. Cooperativity modulates force recovery during consecutive stretches, addressing a key feature missing in the earlier Blum 2020 model.

We incrementally added in each of these features to show their effect on the intrafusal fibre force and consequently the muscle spindle receptor potential. We only tuned *k*_coop_ and used default values for the rest, based on extrafusal thin filament properties, to show the effect of these mechanisms on muscle spindle receptor potential, and therefore the need for their addition to the model.

**FIGURE 2:**
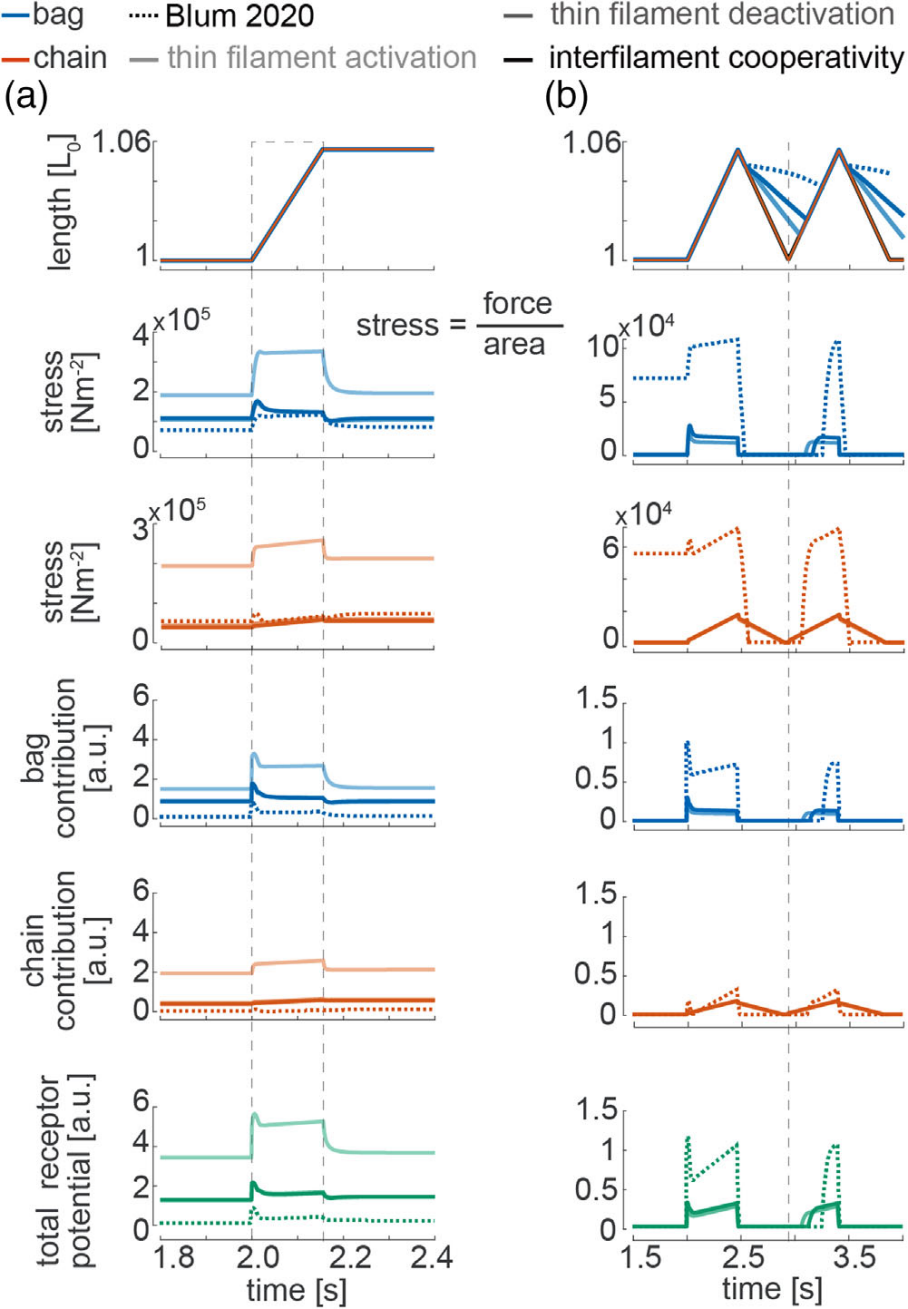
Effect of thin filament dynamics on intrafusal fibre stretch response and muscle spindle receptor potentials. (a) The top row shows ramp‐and‐hold length changes applied to the bag (blue) and chain (red) intrafusal fibres overlaid on top of each other. Dashed box highlights the portion of the protocol when length is changing. Row 2 shows the corresponding stress measured from the bag fibres in blue, and row 3 shows that from the chain fibre in red. From lightest to darkest, the three lines correspond to intrafusal fibres that have (i) thin filament with calcium‐mediated activation, (ii) thin filament that can also deactivate at a slow rate, and (iii) thin filament whose activation also depends on a cooperative interaction with myosin. Since cooperativity has a very small effect, the darkest two lines overlap each other with only the darkest being visible. Dotted lines show the response from the Blum 2020 model, which had no thin filament dynamics. As the lines get darker, the bag fibre exhibits a brief, sharp stress at onset of stretch while the brief, sharp stress in the chain disappears. (b) Top row shows the two triangular stretch–shorten cycles applied to the bag and chain fibres to study the effect of interfilament cooperativity. Lines are colour‐coded similar to (a). Effect of thin filament activation is left out for visual clarity. Dashed vertical line marks start of the second stretch. Slower recovery of stress in the bag fibre on addition of cooperativity can be visualized by the time between the dashed line and stress appearance in row 2. Note that we use different y‐axis scales for the bag and chain fibre stresses to highlight the features of the stress profile. Last three rows in both columns show the receptor potential contribution from the bag, chain and total receptor potential predicted at the muscle spindle spike initiating zone.

We used two protocols to test the effects of thin filament dynamics on the force from bag and chain, and the resulting receptor potential (Figure 2, row 1). The muscle was first activated at pCa 6.4 and held steady for 2 s in both protocols. For the first ramp‐and‐hold protocol (Figure 2a, row 1), we then applied a stretch of velocity 36% *L*_0_ s^−1^ and amplitude 5.6% *L*_0_, where *L*_0_ is the resting length of the half‐sarcomere (Table 1). We chose these values to match the stretch of anaesthetized rat soleus muscle from Housley et al. (2023), to which we qualitatively matched our output (Housley et al., 2023). Once the length reached the maximum value, the fibre was held at the new length for the remainder of the 3 s protocol. We repeated this protocol with the model as we incrementally added the three thin filament properties. The second protocol we used to study the effect of the thin filament dynamics was a stretch–shorten protocol because the third property, cooperativity, modulates force recovery – an effect clearer following a shortening of the intrafusal fibre. For this protocol, we applied a stretch of velocity 12% *L*_0_ s^−1^ and amplitude 5.6% *L*_0_ at 2 s, immediately followed by a shortening of the same velocity and amplitude, and then repeated the stretch–shorten cycle (Figure 2b, row 1). We selected the slower velocity to be able to compare the results to the previous model (Blum et al., 2020). We compared the model's response to both protocols to that from the Blum 2020 model (Figure 2).

### 2.5 Effect of thick filament dynamics on force response to stretch

Using the same ramp‐and‐hold protocol as above, we tested the effect of varying myosin dynamics. To limit the number of free parameters in the model, we selected one parameter per rate function to test the effect of the thick filament interaction with the added thin filament in modulating force, and therefore, receptor potential. We systematically varied the myosin attachment rates (*b*_f_, *c*_f_) and detachment rates (*b*_g_, *c*_g_) in the intrafusal fibres through three orders of magnitude.

### 2.6 Tuning model parameters to match experimental data

**FIGURE 3:**
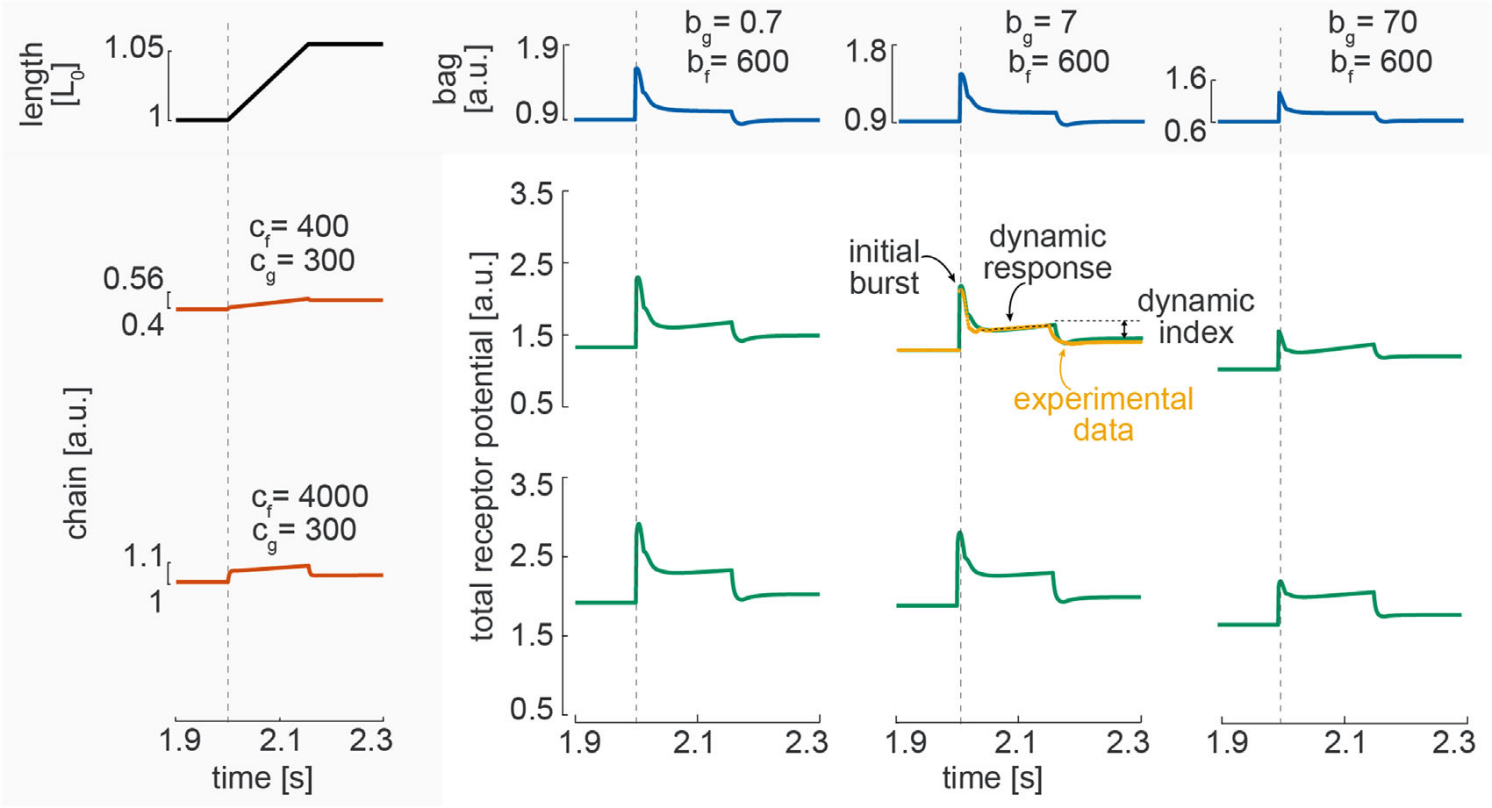
Model‐predicted receptor potential for six sets of attachment and detachment rates of the bag and chain fibres. The top left corner shows the ramp‐and‐hold length change protocol used for all conditions. The rest of the top row and first column show the contribution from the bag (blue) and chain fibres (red) to the total receptor potential (green), respectively. For clarity of presentation, we show the effect of three detachment rates of the bag fibre while keeping its attachment rate fixed; detachment rate increases from left to right, and we show the effect of two attachment rates of the chain fibre while keeping its detachment rate constant; and attachment rate increases from top to bottom. Each increase is by an order of magnitude as indicated by *b*_g_ (bag detachment rate) and *c*_f_(chain attachment rate). The yellow dots are data of instantaneous firing rate collected from an anaesthetized rat soleus undergoing a similar protocol (Housley et al., 2023). The baseline firing rate of the experimental data was shifted up to align with that of the model trace in green.

We matched qualitative features of our model's response to the ramp‐and‐hold protocol to experimentally measured instantaneous firing rate from literature (Figure 3). We used published data of a single trial where an adult rat was anaesthetized and the Ia afferent firing in response to its soleus being stretched was measured at the axon in its dorsal root (Housley et al., 2023). We tuned *b*_f_, *c*_f_, *b*_g_ and *c*_g_ to match the following three features. We quantified these features as defined below to emphasize the effects of the parameters on the features. However, we did not use those quantities to optimize the parameters as we were only interested in understanding the effects of the parameters on the features in this study.

- A high *initial burst* at the onset of stretch: magnitude of the first peak in receptor potential after subtracting the baseline receptor potential pre‐stretch.
- A *dynamic response* where the receptor potential rises during stretch after initial burst: slope of the receptor potential during stretch after initial burst. We used the *polyfit* command in MATLAB 2022b (The MathWorks, Natick, MA, USA) to fit a line to the receptor potential from its lowest value after initial burst till the end of ramp.
- A steady‐state response during the hold phase where the receptor potential is lower than that at the end of the ramp, and characterized by the *dynamic index*: the difference between the magnitude of the peak dynamic response and final receptor potential during hold‐phase.

### 2.7 Predicting history dependence in stretch–shorten cycles

We used three protocols to characterize the model's history‐dependence.

#### 2.7.1 Time history dependence in triangular stretch–shorten cycles

Two triangular stretch–shorten cycles were applied with varying inter‐stretch intervals (ISI) to evaluate the effect on the second stretch–shorten cycle. At 2 s, we applied the *conditioning stretch*, at velocity 12% *L*_0_ s^−1^ and amplitude 5.6% *L*_0_, where *L*_0_ is the resting length of our half‐sarcomere model. This matches the protocol used to evaluate the thin filament dynamics described earlier. Once the length reached its maximum value, we shortened the fibre by the same velocity until it reached its initial length. Then, the *test stretch* consisted of the same stretch–shorten cycle applied after a range of ISIs from 0 s to10 s. We report the ISI required for the initial burst to reappear in the test stretch, its magnitude, the ISI required for the initial burst to reach the same magnitude as that of the conditioning stretch, and its comparison to literature.

#### 2.7.2 Amplitude history dependence in triangular stretch–shorten cycles

Two triangular stretch–shorten cycles were applied exactly as those used to test the effect of ISI. However, the ISI was kept fixed at 0 s and instead the amplitude of the conditioning stretch was varied to evaluate its effect on a second stretch–shorten cycle. We used a range of conditioning stretch amplitude from 0% *L*_0_ to 5.6% *L*_0_ and kept the second test stretch fixed at an amplitude of 5.6% *L*_0_. We report the conditioning amplitude that eliminates the initial burst in the test stretch.

#### 2.7.3 History dependence and non‐linearities in sinusoids

We used three sinusoidal stretch–shorten cycles of amplitudes ranging from 0.0016% *L*_0_ to 1.6% *L*_0_ at a frequency of 1 Hz to test history dependence in the first and second stretch, and non‐linearities in response to sinusoid amplitude reported previously (Matthews & Stein, 1969).

## 3 RESULTS

### 3.1 Thin filament dynamics differentially affect bag and chain fibres

During ramp and hold stretches, adding thin filament dynamics had opposing effects on the force at stretch onset in the bag versus chain fibre. In the bag fibre, adding calcium‐dependent activation alone did not change the qualitative features of the force (Figure 2a, row 2, dotted vs. lightest blue). The further addition of a deactivation rate to the actin sites led to a sharp, brief increase in force at stretch onset in the bag fibre (Figure 2a, row 2, lightest vs. darkest blue). In contrast, adding thin filament dynamics to the chain fibre eliminated the previously present sharp, brief rise in force (Figure 2a, row 3, dotted vs. lightest red) while the addition of the deactivation rate did not qualitatively change the force (Figure 2a, row 3, lightest vs. darkest red). As suspected, adding cooperativity between the thin and thick filaments did not qualitatively change the force profile in the bag or chain fibre. This can be observed by the two darkest lines (Figure 2a, row 2 blue, row 3 red) nearly overlapping each other.

During repeated triangular stretch–shorten cycles, the force recovery after a shortening was faster in both fibres when adding the thin filament, and slowed by increasing cooperativity only in the bag fibre (Figure 2b). Adding the thin filament reduced the time of force recovery in the second, test stretch by 50% from 313 ms to 158 ms after stretch onset in the bag fibre (Figure 2b, row 2, dotted vs. dark blue, 2nd cycle) and by 98%, from 118 ms to 2 ms after stretch onset in the chain fibre (Figure 2b, row 3, dotted vs. light red, 2nd cycle). Adding *k*_coop_ = 1 delayed the recovery by 42% from 158 ms to 224 ms in the bag fibre (Figure 2b, row 2, light vs. dark blue, 2nd cycle) but did not affect the force recovery time of the chain fibre (Figure 2b, row 3, light vs. dark red, 2nd cycle).

Simulated receptor potentials in ramp and hold stretches were also affected by adding thin filament dynamics to bag and chain fibre forces, altering their relative contributions to receptor potential features. The initial bag response resembled that of the ramp acceleration (Figure 2a, row 4, Figure 2b row 4, 1st cycle) and the chain response resembled that of the ramp stretch displacement (Figure 2a row 5, Figure 2b row 5, 1st cycle). While this is similar to the Blum 2020 model, the relative contribution of the components ($k_{f_{b}}$, $k_{y_{b}}$, $k_{f_{c}}$) is different. We use $k_{f_{b}}$ = 0.4, $k_{y_{b}}$ = 0.005 and $k_{f_{c}}$ = 0.5 here while Blum 2020 used $k_{f_{b}}$ = 1, $k_{y_{b}}$ = 0.03 and $k_{f_{c}}$ = 1 (Table 1). Since the absolute values of these weightings are not directly comparable between models, the notable point is that the bag yank contributes much less in this model relative to the bag and chain force, and the chain force contributes slightly more than the bag force here, while the two made an equal contribution in the Blum 2020 model. This suggests that the thin filament dynamics of the bag fibre can encode the yank‐like component that leads to the initial rise in receptor potential in the force.

Finally, the time course of the receptor potential was most similar to the time course of force recovery in the bag fibre (Figure 2b, row 6). Addition of the thin filament led to the receptor potential on the test stretch reappearing 59% faster from 316 ms to 130 ms faster (Figure 2b, row 5, dotted vs. light green). Adding cooperativity between the filaments delayed the recovery by 49% from 130 ms to 194 ms (Figure 2b, row 6, light vs. dark green) which was still faster by 39% (316 ms vs. 194 ms) than the Blum model (Figure 2b, row 6, dark vs. dotted green). For all subsequent simulations, we used *k*_coop_ = 1.

### 3.2 Myosin dynamics qualitatively alter bag and chain fibre response to stretch

During ramp and hold stretches, varying myosin attachment and detachment rates in the bag and chain fibres affected three key features of simulated receptor potentials. Given the large number of combinations possible with four parameters, we present only six combinations here. The receptor potential has low sensitivity to small changes in rates, and varying only bag detachment rate or chain attachment had negligible effects on receptor potential. Thus, we focus on order of magnitude changes in the bag attachment rate and chain detachment rate. We present three values of bag attachment rate (*b*_f_) while keeping the detachment rate (*b*_g_) constant, and two values of chain detachment rate (*c*_g_) while keeping the attachment rate (*c*_f_) constant during a ramp and hold stretch (Figure 3).

The initial burst arose from the interaction between bag and chain fibres (Figure 3, row 2 and 3, green). Initial burst non‐linearly decreased by 9% from 0.97 to 0.88 for an order of magnitude increase in bag fibre detachment rate *b*_g_ (Figure 3, row 2, column 2 vs. 3, green) but further increasing it by another order of magnitude decreased the initial burst by 40% to 0.53 (Figure 3, row 2, column 3 vs. 4, green). This effect was consistent even when the chain fibre detachment rate was increased by an order of magnitude – the initial burst first decreased by 2% from 1.09 to 1.01 and then dropped by 40% to 0.61 (Figure 3, row 3, column 2 vs. 3 and column 3 vs. 4 green). However, the overall increased force due to the chain fibre, and consequent peak dynamic response (observed through increased dynamic index with increasing *c*_f_) could obscure the initial burst (Figure 3, column 3, row 2 vs. 3, green).

The dynamic response was primarily modulated by the bag fibre detachment rate. Increasing the bag fibre detachment rate caused the bag force to decrease (Figure 3, row 1, blue, left to right), had a non‐linear effect of making the dynamic response of the receptor potential more prominent, and had no effect on the dynamic index. The total receptor potential reflected more chain‐like behaviour – increasing more sharply during stretch, that is, exaggerated dynamic response (Figure 3, row 2 and 3, green, left to right). The dynamic response first increased by 2% from 0.81 to 0.83 for an order of magnitude change of *b*_g_ (Figure 3, row 2, column 2 vs. 3, green). Further increasing *b*_g_ by another order of magnitude increased the dynamic response by 18% to 0.98 (Figure 3, row 2, column 3 vs. 4, green). Similarly, for another set of chain fibre attachment rates, the dynamic response followed the same trend of increasing by 4% from 0.47 to 0.49 and then by 31% to 0.64 (Figure 3, row 2, column 2 vs. 3 and 3 vs. 4, green).

The dynamic index was primarily modulated by the chain fibre attachment rate. Increasing the attachment rate of the chain fibre while maintaining its detachment rate caused the chain fibre to have an overall increased force but a shallower slope through the stretch, leading to an increased dynamic index in the receptor potential (Figure 3, row 2 vs. 3, green). An order of magnitude increase in *c*_f_ consistently lowered the dynamic response but the magnitude of reduction was dominated by the bag fibre rates as described in the previous paragraph. The dynamic index, however, consistently nearly doubled for three different bag fibre detachment rates (0.18 to 0.34, 0.19 to 0.34 and 0.17 to 0.32) for the same order of magnitude increase in *c*_f_.

### 3.3 Predicting history dependence in muscle receptor potentials

For history dependence predictions, we selected the myosin parameters that best matched the data of instantaneous firing rate from a rat soleus muscle (Figure 3; row 2, column 3, green vs. yellow; *b*_f_ = 600 s^−1^, *b*_g_ = 7 s^−1^, *c*_f_ = 400 s^−1^ and *c*_g_ = 300 s^−1^). This set of parameters matched features of the initial burst, dynamic response, and dynamic index as described under the section ‘Tuning model parameters to match experimental data’.

**FIGURE 4:**
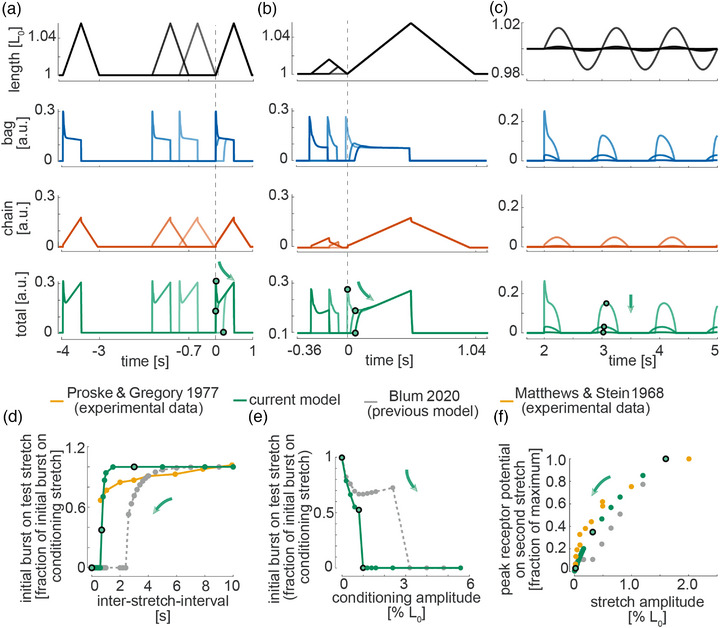
Model predictions of history dependence during cyclic movements. In (a–c), the top row shows the length changes in black, the second row shows the bag contribution to the total receptor potential in blue, the third row shows the same from the chain fibre in red, and the fourth row shows the total receptor potential in green. (a) We subjected the model to two consecutive triangular stretch–shorten cycles where the inter‐stretch interval was varied between 0 s and 10 s. For clarity, we only show three inter‐stretch intervals of 0 s, 0.7 s and 3 s (lightest to darkest) representing when the initial burst disappears, just appears and reachs maximum magnitude. Black circles in row 4 highlight the initial burst magnitude that is plotted against the inter‐stretch interval in (d). There is no initial burst in the test stretch of the 0 s ISI (lightest) condition but it returns to maximum value by 3 s (darkest). (b) We repeated the two triangular stretch–shorten cycles with an inter‐stretch‐interval of 0 s but varied the amplitude of the first test stretch from 0% *L*_0_, to 5.6% *L*_0_ (lightest to darkest). There is an initial burst at the onset of stretch in the 0% *L*_0_ (lightest) condition which disappears after 0.8% *L*_0_ (darkest). Black circles in row 4 highlight the initial burst magnitude that is plotted against the conditioning amplitude in (e). The traces in (a) and (b) are time‐aligned such that 0 s is when the test stretch begins. (c) We subjected the model to three cycles of sinusoidal stretch–shorten cycles of a range of amplitudes from 0% *L*_0_ to 1.6% *L*_0_ at 1 Hz. For visual clarity, we show results from three of those amplitudes that are an order of magnitude apart: 0.016% *L*_0_, 0.16% *L*_0_, 1.6% *L*_0_. Length changes phase lag the receptor potential, and only the first cycle shows an initial burst, as qualitatively estimated by the sharp rise in receptor potential (bottom row, green) at the onset of first stretch, which reduces with decreasing stretch amplitude, and the magnitude of receptor potential is always lower on the second cycle. (f) Magnitude of the peak receptor potential on the second stretch cycle non‐linearly decreases with the amplitude of the sinusoidal stretch.

For two consecutive triangular stretch–shorten cycles, the simulated receptor potential in the second, test stretch progressively reduced as the inter‐stretch time interval decreased. The initial burst was abolished at ISIs less than 0.7 s and the dynamic response was delayed due to the slackening of the intrafusal fibres (Figure 4a). This is 4.3 times faster than the prior model (Blum et al., 2020), which required ∼3 s of ISI for the initial burst to start reappearing on the test stretch (Figure 4d). For visual clarity, we only show the time series results for the 0 s, 0.7 s and 3 s ISIs (Figure 4a). The initial burst magnitude was reduced to 40% of the initial conditioning stretch after an ISI of 0.7 s (Figure 4d) and returned completely by ISIs of 3 s (Figure 4a,d). This is comparable to that observed in literature (Proske & Gregory, 1977) where the initial burst on the test stretch was 60% of the conditioning stretch after 0.5 s and returned completely by 10 s (Figure 4d).

When conditioning stretch amplitude was systematically varied, there was a similar modulation of the initial burst and dynamic response in the second, test stretch. The two cycles always occurred with inter‐stretch interval of 0 s (Figure 4b, row 1). As the conditioning stretch amplitude increased, the magnitude of the initial burst decreased until the conditioning stretch amplitude reached 0.8% *L*_0_, when the initial burst was 52% of the unconditioned stretch (Figure 4b row 4, 4e). The initial burst in the test stretch was abolished for larger conditioning stretch amplitudes (Figure 4e).

In sinusoidal stretches, the model predicted the larger muscle spindle responses in the first stretch cycle as well as non‐linear reductions in steady‐state receptor potential with increasing stretch amplitude. The first stretch cycle had a qualitatively different receptor potential compared to later cycles, exhibiting initial bursts at the onset of stretch (Figure 4c, row 4). The second and third cycles were of lower amplitude, and resembled the half‐wave rectified version of the changes in length, with a phase advance (Figure 4c) as reported in the literature (Abbot et al., in review; Day et al., 2017; Matthews & Stein, 1969). Finally, the muscle spindle model also predicted non‐linearities in the response of the muscle spindle receptor potential to sinusoidal stretch amplitude. The amplitude of the peak receptor potential of the second and third stretches increased non‐linearly with stretch amplitude, with a steep slope at smaller amplitudes, and a shallower slope at higher amplitudes (Figure 4f); this behaviour has been reported previously in the literature (Matthews & Stein, 1969).

## 4 DISCUSSION

In a biophysical muscle spindle model, we show that adding actin dynamics – and its consequent interaction with myosin dynamics – improved predictions of history‐dependent muscle spindle firing. Our model augments that presented by Blum et al. (2020) by adding actin dynamics of the thin filament that interacts with the myosin dynamics of the existing thick filament model. We show how these interactions alter intrafusal muscle fibre forces, providing a platform for testing the role of cross‐bridge properties in shaping muscle spindle firing responses. Importantly, adding actin dynamics improved the history‐dependent behaviour of the model to be more in line with experimental observation. Initial bursts evoked by stretch–shorten cycles are eliminated by a prior stretch–shorten cycle and begin to reappear as the ISI reaches about 0.7 s, 4.3 times faster than the prior model. The model further predicts that muscle spindle stretch responses are increasingly attenuated based on the amplitude of an immediately preceding stretch–shorten cycle. Importantly, the model predicts greater muscle spindle responses in the first compared to later sinusoidal stretch cycles, as well as a non‐linear steady‐state firing response to sinusoid amplitude, both shown experimentally in the literature. As such the present model demonstrates how a variety of non‐linear and history‐dependent muscle spindle firing behaviours emerge from intrafusal cross‐bridge dynamics. Further, the implementation of the model on an open‐source, validated muscle modelling platform facilitates reproducibility and broader use of the model for linking biophysical properties of intrafusal fibres to muscle spindle function.

We show that differences in the intrafusal fibre contractile properties can give rise to differences in muscle spindle response comparable to those attributed to the other regions of the muscle spindle. In contrast, the Blum model – the only other muscle spindle model to simulate intrafusal cross‐bridge dynamics – focused on varying mechanotransduction sensitivities between the central encoding region to the Ia afferent membrane (Bewick & Banks, 2021) of simulated bag and chain fibre forces; parameter values of the bag and chain fibres were fixed. However, these intrafusal fibres vary structurally in the type of myofibrillar ATPase due to their molecular composition of different myosin isoforms that affect cross‐bridge dynamics and therefore contraction of the muscle fibre. Bag1 intrafusal fibres give rise to highly dynamic and history‐dependent firing features of muscle spindle Ia afferents and have slower contractile properties than the bag2 and chain fibres. Bag2/chain intrafusal fibres produce more static components of firing in muscle spindle Ia and group II afferents due to their faster contractile properties (Banks, 2005; Poppele & Quick, 1985, 1981). Intrafusal fibres also vary widely in both structure and function across species, ageing and pathologies (Macefield & Knellwolf, 2018; Thornell et al., 2015). However, few studies have demonstrated the functional differences in muscle spindle firing caused by changes in the biophysical properties of the intrafusal fibres (Cazzato & Walton, 1968; Papaioannou & Dimitriou, 2020). Here we show that the firing properties can be tuned by altering actin–myosin interactions, producing a spectrum of muscles spindle responses to stretch. However, muscle spindle firing is also affected by the mechanotransduction processes in the central encoding region (Blum et al., 2020) as well as neuronal firing dynamics that can also change in pathology (Housley et al., 2023). Further insight to muscle spindle firing properties could be gained through modelling all of these mechanisms.

Once tuned, intrafusal cross‐bridge dynamics predicted a variety of history‐dependent and non‐linear properties of muscle spindle firing, including those in sinusoidal stretches. History‐dependent muscle spindle firing responses have most commonly been investigated based on the disappearance and recovery of the initial burst by increasing the rest time between two stretch–shorten cycles. This property has long been hypothesized to be due to the short‐range stiffness of muscle spindle intrafusal fibres (Haftel et al., 2004; Hasan & Houk, 1975; Poppele & Quick, 1985, 1981), and simulated recently in the simpler version of our biophysical muscle spindle model (Blum et al., 2020). Short range stiffness manifests as a large, transient force at the onset of stretch when the attached cross‐bridges are pulled until they detach (Campbell & Moss, 2002; Lakie & Campbell, 2019; Rack & Westbury, 1974). Here we show that differences in actin and myosin dynamics in the intrafusal fibres govern the degrees and temporal features of intrafusal short‐range stiffness by altering how quickly cross‐bridges form and break. In particular, adding actin dynamics increased the amount of short‐range stiffness in the bag1 fibre and how quickly it recovered. As the number of attached cross‐bridges determines the force upon stretch, and is shaped by the history of length, velocity and activation of the muscle fibre, there are many ways that muscle spindle history dependence can manifest in complex stretch profiles. We show that the same cross‐bridge dynamics giving rise to time‐history dependence also predicts amplitude‐history dependence (Hasan & Houk, 1975; Hunt & Ottoson, 1975; Huyghues‐Despointes et al., 2003), as well as history dependence in sinusoidal stretches (Abbot et al., in review; Day et al., 2017; Matthews & Stein, 1969). Importantly, the model not only predicts greater firing in the first sinusoidal stretch, but also predicts greater sensitivity of steady‐state muscle spindle firing to smaller versus larger stretch amplitudes shown previously (Matthews & Stein, 1969).

The history‐dependent and non‐linear properties of muscle spindle stretch response shown here have implications for the control of posture and movement, particularly standing balance control (Ivanenko & Gurfinkel, 2018). Muscles are constantly being stretched and shortened during quiet standing at about 1 Hz, with muscle stretch amplitude estimated to be less than 1% *L*_0_. However, in balance‐impaired individuals, sway frequency increases, and sway amplitude can reach up to 3% *L*_0_. Our simulations show that the muscle spindle firing during larger sinusoids attenuates at greater amplitudes, attenuating the sensory information conveyed. Further, in response to a sudden perturbation, our model predicts that the muscle spindle initial bursts are nearly eliminated by stretches greater than 0.8% *L*_0_ but maintained at smaller stretches. The muscle spindle initial burst is hypothesized to play a critical role in signalling the onset of a disturbance and enabling a rapid sensorimotor response for balance correction (D. C. Lin et al., 2019; Sober et al., 2018), and is absent in animals with balance deficits due to loss of group I sensory afferents (Lockhart & Ting, 2007). Overall, our simulations suggest that the conditions of increased postural sway with balance impairments may cause reduced muscle spindle response to stretch, decreasing the subsequent sensorimotor response. Further simulations with the muscle spindle model in parallel with an activated extrafusal muscle fibre, and modulated with varying gamma drive to the intrafusal fibre can be used to test these predictions under more realistic postural sway conditions.

Using a validated, open‐source platform for simulating muscle cross‐bridge dynamics facilitates the broader use and application of our muscle spindle model to link muscle spindle biology to its physiological function. The MATMyoSim platform hosts a variety of more complex biophysical muscle properties, enabling more complexity to be more easily added (Campbell, 2014). Nonetheless, the cross‐bridge model presented here is complex enough to generate physiologically realistic responses to both discrete and continuous perturbations, with the ability to robustly simulate sinusoids being an advance over the prior model. MATMyoSim's model and protocol definition files also enable better reproducibility and comparisons across simulations and across users, whereas the prior model required changes in the simulation code itself. While MATMyosim is primarily used to test effects of muscle length changes on muscle fibre mechanics, it also has the capability to simulate muscle fibres in closed‐loop simulations with external loads, potentially enabling muscle spindle function during simulated movements to be investigated. As such, the model presented here can serve as an open platform for simulating muscle spindle function and the role of intrafusal contractile properties on muscle spindle function in health and disease.

## AUTHOR CONTRIBUTIONS

Surabhi N. Simha and Lena H. Ting conceived and designed the project. Surabhi N. Simha obtained the results. Surabhi N. Simha and Lena H. Ting interpreted the results. Surabhi N. Simha and Lena H. Ting drafted and revised the final version of the manuscript. All authors have read and approved the final version of this manuscript and agree to be accountable for all aspects of the work in ensuring that questions related to the accuracy or integrity of any part of the work are appropriately investigated and resolved. All persons designated as authors qualify for authorship, and all those who qualify for authorship are listed.

## CONFLICT OF INTEREST

The authors declare no conflicts of interest.

## Data Availability

No data were collected for this study. Code for the model is publicly available at https://github.com/Neuromechanics‐Lab/Simha‐Ting2023JExptPhysiol.
